# Different epidemiology of bloodstream infections in COVID-19 compared to non-COVID-19 critically ill patients: a descriptive analysis of the Eurobact II study

**DOI:** 10.1186/s13054-022-04166-y

**Published:** 2022-10-18

**Authors:** Niccolò Buetti, Alexis Tabah, Ambre Loiodice, Stéphane Ruckly, Abdullah Tarik Aslan, Giorgia Montrucchio, Andrea Cortegiani, Nese Saltoglu, Bircan Kayaaslan, Firdevs Aksoy, Akova Murat, Özlem Akdoğan, Kemal Tolga Saracoglu, Cem Erdogan, Marc Leone, Ricard Ferrer, José-Artur Paiva, Yoshiro Hayashi, Mahesh Ramanan, Andrew Conway Morris, François Barbier, Jean-François Timsit, the Eurobact 2 study group, Jeffrey Lipman, Edward Litton, Anna Maria Palermo, Timothy Yap, Ege Eroglu, Koji Hosokawa, Hideki Yoshida, Shigeki Fujitani, Farid Zand, Ata Mahmoodpoor, Seyed Mohammad Nasirodin Tabatabaei, Omar Elrabi, Ghaleb A. Almekhlafi, Gabriela Vidal, Marta Aparicio, Irene Alonzo, Silvio A. Namendys-Silva, Mariana Hermosillo, Roberto Alejandro Castillo, Liesbet De Bus, Jan De Waele, Isabelle Hollevoet, Nicolas De Schryver, Nicolas Serck, Pedja Kovacevic, Biljana Zlojutro, Etienne Ruppe, Philippe Montravers, Thierry Dulac, Jérémy Castanera, Alexandre Massri, Charlotte Guesdon, Pierre Garcon, Matthieu Duprey, François Philippart, Marc Tran, Cédric Bruel, Pierre Kalfon, Gaëtan Badre, Sophie Demeret, Loïc Le Guennec, Matteo Bassetti, Daniele Giacobbe, Gabriele Sales, Ivan Daroui, Giovanni Lodi, Mariachiara Ippolito, Davide Bellina, Andrea Di Guardo, Monica Rocco, Silvia Fiorelli, Adam Mikstacki, Mariusz Peichota, Iwona Pietraszek-Grzywaczewska, Pedro Póvoa, Andriy Krystopchuk, Ana Teresa, António Manuel Pereira de Figueiredo, Isabel Botelho, Vasco Costa, Rui Pedro Cunha, Alexey Gritsan, Vladislav Belskiy, Mikhail Furman, Maria Martinez, Vanessa Casares, Maria Pilar Gracia Arnillas, Rosana Munoz Bermudez, Alejandro Ubeda, Maria Salgado, Emilio Maseda, Alejandro Suarez De La Rica, Miguel Angel Blasco-Navalpotro, Alberto Orejas Gallego, Josef Prazak, J. L. Pagani, S. Abed-Maillard, Arzu Topeli Iskit, Selcuk Mehtap, Solakoğlu Ceyhun, Ayşe Kaya Kalem, Ibrahim Kurt, Murat Telli, Barcin Ozturk, Nurcan Baykam, Ridvan Karaali, Iftihar Koksal, Yeliz Bilir, Seda Guzeldag, Gulden Ersoz, Guliz Evik, Yasar Bayindir, Yasemin Ersoy, Ari Ercole, Ashok Raj, Artemis Zormpa, George Tinaslanidis, Reena Khade, Ashraf Roshdy, Santhana Kannan, Supriya Antrolikar, Nicholas Marsden, Ben Attwood, Jamie Patel, Mohan Gurjar, Carol Dsilva, Jagadish Chandran, Bashir El Sanousi, Elfayadh Saidahmed, Hytham K. S. Hamid

**Affiliations:** 1 Infection Control Program and WHO Collaborating Centre on Patient Safety, Geneva University Hospitals and Faculty of Medicine, Rue Gabrielle-Perret-Gentil 4, 1205 Geneva, Switzerland; 2 INSERM, IAME, Université Paris-Cité, 75006 Paris, France; 3 Intensive Care Unit, Redcliffe Hospital, Metro North Hospital and Health Services, Brisbane, QLD Australia; 4 Queensland University of Technology, Brisbane, QLD Australia; 5 Faculty of Medicine, University of Queensland, Brisbane, QLD Australia; 6 ICURESEARCH, 26 rue Garibaldi, Fontaine, France; 7 Department of Internal Medicine, Hacettepe University, Sihhiye, Ankara, Turkey; 8 Department of Surgical Sciences, University of Turin, Turin, Italy; 9 Department of Anaesthesia, Critical Care and Emergency, Città Della Salute e Della Scienza Hospital, Turin, Italy; 10 Department of Surgical Oncological and Oral Science (Di.Chir.On.S.), University of Palermo, Palermo, Italy; 11 Department of Anesthesia, Intensive Care and Emergency, Policlinico Paolo Giaccone, Palermo, Italy; 12 Department of Infectious Diseases and Clinical Microbiology, Istanbul University-Cerrahpasa, Istanbul, Turkey; 13 Department of Infectious Diseases and Clinical Microbiology, Ankara City Hospital, Ankara Yıldırım Beyazıt University, Ankara, Turkey; 14 Department of Infectious Diseases and Clinical Microbiology, Karadeniz Technical University, Ortahisar, Turkey; 15 Department of Infectious Diseases and Clinical Microbiology, Hacettepe University, Ankara, Turkey; 16 Department of Infectious Diseases and Clinical Microbiology, Erol Olçok Research and Training Hospital, Hitit University, Çorum Merkez, Turkey; 17 Department of Anesthesiology and Reanimation, Kartal Dr. Lütfi Kırdar City Hospital, Kartal, Turkey; 18 Department of Anesthesiology and Reanimation, Medipol Mega Hospital, Bağcılar, Turkey; 19 Department of Anesthesiology and Intensive Care Unit, Hospital Nord, Assistance Publique Hôpitaux Universitaires de Marseille, Aix Marseille University, Marseille, France; 20 Intensive Care Department. SODIR Research Group, Vall d’Hebron Institute of Research VHIR, Hospital Universitari Vall d’Hebron, Barcelona, Spain; 21 Intensive Care Medicine Department, Centro Hospitalar Universitário São João (CHUSJ), Porto, Portugal; 22 Department of Medicine, Faculty of Medicine, University of Porto (FMUP), Porto, Portugal; 23 Department of Intensive Care Medicine, Kameda General Hospital, Kamogawa, Japan; 24 Caboolture and The Prince Charles Hospitals, Metro North Hospital and Health Services, Brisbane, QLD Australia; 25 Critical Care Division, The George Institute for Global Health, University of New South Wales, Sydney, Australia; 26 School of Medicine, University of Queensland, St Lucia, Australia; 27 Division of Anaesthesia, Department of Medicine, University of Cambridge, Cambridge, UK; 28 Division of Immunology, Department of Pathology, University of Cambridge, Cambridge, UK; 29 JVF Intensive Care Unit, Addenbrooke’s Hospital, Cambridge, UK; 30 Médecine Intensive Réanimation, Centre Hospitalier Régional d’Orléans, Orléans, France; 31 Centre d’Étude des Pathologies Respiratoires (CEPR), INSERM U1100, Université de Tours, Tours, France; 32 Medical and Infectious Diseases Intensive Care Unit, AP-HP, Bichat-Claude Bernard University Hospital, 46 rue Henri Huchard, 75877 Paris Cedex, France

**Keywords:** Bloodstream infection, ICU-acquired, COVID-19, *Enterococcus*, Bacteremia

## Abstract

**Background:**

The study aimed to describe the epidemiology and outcomes of hospital-acquired bloodstream infections (HABSIs) between COVID-19 and non-COVID-19 critically ill patients.

**Methods:**

We used data from the Eurobact II study, a prospective observational multicontinental cohort study on HABSI treated in ICU. For the current analysis, we selected centers that included both COVID-19 and non-COVID-19 critically ill patients. We performed descriptive statistics between COVID-19 and non-COVID-19 in terms of patients’ characteristics, source of infection and microorganism distribution. We studied the association between COVID-19 status and mortality using multivariable fragility Cox models.

**Results:**

A total of 53 centers from 19 countries over the 5 continents were eligible. Overall, 829 patients (median age 65 years [IQR 55; 74]; male, *n* = 538 [64.9%]) were treated for a HABSI. Included patients comprised 252 (30.4%) COVID-19 and 577 (69.6%) non-COVID-19 patients. The time interval between hospital admission and HABSI was similar between both groups. Respiratory sources (40.1 vs. 26.0%, *p* < 0.0001) and primary HABSI (25.4% vs. 17.2%, *p* = 0.006) were more frequent in COVID-19 patients. COVID-19 patients had more often enterococcal (20.5% vs. 9%) and *Acinetobacter* spp. (18.8% vs. 13.6%) HABSIs. Bacteremic COVID-19 patients had an increased mortality hazard ratio (HR) versus non-COVID-19 patients (HR 1.91, 95% CI 1.49–2.45).

**Conclusions:**

We showed that the epidemiology of HABSI differed between COVID-19 and non-COVID-19 patients. Enterococcal HABSI predominated in COVID-19 patients. COVID-19 patients with HABSI had elevated risk of mortality.

*Trial registration* ClinicalTrials.org number NCT03937245. Registered 3 May 2019.

**Supplementary Information:**

The online version contains supplementary material available at 10.1186/s13054-022-04166-y.

## Background

Hospital-acquired bloodstream infections (HABSI) are a frequent event in critically ill patients and are associated with increased morbidity and mortality [1, 2]. Severe acute respiratory syndrome coronavirus 2 (SARS-CoV-2) virus emerged in 2019 and its disease (COVID-19) caused millions of deaths worldwide. Probably due to various reasons, critically ill patients infected with SARS-CoV-2 were more prone to hospital-acquired infections [3], more specifically bloodstream infections (BSIs) [4]. A recent systematic review showed a pooled estimated occurrence of BSIs of almost 30% in patients admitted to intensive care unit (ICU) [5]. Several epidemiological studies suggested that HABSI acquired in the ICU occurred more often during the different COVID-19 waves [6, 7]. Multicentric analyses illustrated that ICU-BSI in COVID-19 patients were associated with prolonged length of ICU stay and increased mortality [8].

Most of the literature has focused on COVID-19 patients and little is known about differences in the pathogen distribution between COVID-19 and non-COVID-19 patients. In August 2019, we started the Eurobact II study which included critically ill ICU patients with HABSIs, regardless of their status with respect to COVID-19 infection. The data collection was continued during the different COVID-19 waves, thus allowing an accurate evaluation of the epidemiology of HABSIs in ICU patients during the study period. The primary objective of this study was to describe the epidemiology of HABSI between COVID-19 and non-COVID-19 critically ill patients in terms of patients’ characteristics, source of infection, microorganism distribution and mortality.

## Material and methods

### Eurobact II study design

The Eurobact II study was a prospective observational multicontinental cohort study conducted between August 2019 and June 2021 [9]. This observational study was registered within ClinicalTrials.org (NCT03937245) and was reported in accordance with the STrengthening the Reporting of OBservational studies in Epidemiology (STROBE) guidelines [10].

### Setting

Endorsement, financial and logistical support was obtained from the European Society of Intensive Care Medicine (ESICM), the Infectious Diseases (ESCMID) study Group for Infections in Critically Ill Patients (ESGCIP) and the European Society of Clinical Microbiology. An operational committee (AT, NB, FB, SR and JFT) was constituted to oversee all study operations. Study oversight and logistics were provided through a non-profit research organization, the OUTCOMEREA^®^ study group (Paris, France). A Scientific Committee and National coordinators (NCs) were recruited for each participating country by the operational committee with assistance from the endorsing societies. Main responsibilities of NCs included recruiting participating ICUs, applying for ethical and regulatory approvals at national level where applicable, and facilitating communication with ICUs within their countries.

### ICU and patient recruitment

ICUs eligible to participate were defined as a department specifically designed to manage patients with organ failures within a health-care facility and able to provide invasive mechanical ventilation for a duration of at least 24 h. For this observational study, among all Eurobact II participating ICUs, we selected only those that recruited both COVID-19 and non-COVID-19 patients.

Patients ≥ 18 years old with a first episode of HABSI *treated* in ICU were included. HABSI was defined as a positive blood culture sampled 48 h after hospital admission. Both patients with blood cultures sampled in ICU (i.e., ICU-acquired HABSI) and patients transferred (i.e., in 48 h) to the ICU for the treatment of the HABSI were enrolled.

Blood cultures with typical skin contaminants (e.g., coagulase-negative staphylococci, *Corynebacterium* species, *Bacillus* species, *Propionibacterium* species, *Micrococcus* species) were included if at least two blood cultures with the same antimicrobial susceptibility profile were observed or strong clinical grounds that the blood culture was not a contaminant (e.g., infected material proven as a source for the HABSI). All HABSIs including typical skin contaminants were carefully reviewed by at least one member of the scientific committee (AT, FB or NB). Only the first bloodstream infection during the eligibility period was included for the current analysis. We excluded community-acquired bloodstream infections, typical skin contaminants that did not fulfill inclusions criteria, cases with missing core outcome data (i.e., dates of bloodstream infections and hospital/ICU admission, dates of discharge and/or death as applicable, pathogen and treatment inclusive of antimicrobials and source control as applicable) and retrospective inclusions.

The Eurobact II study recruited centers with HABSI from 1st August 2019 to 30th January 2021. The minimal recruitment period was 3 months or 10 consecutive HABSIs (whichever came first) and could be extended on request from the local investigator for the whole duration of the study. Of note, a flexible start of the inclusion period was allowed for each ICU to facilitate participation in the study.

### Data collection

The Eurobact II was an observational study, pre-specifying that all data had to be collected from the patients’ chart without additional diagnostic tests or interventions.

The study website and case report form (CRF) comprised a center form that collected data which described the ICU, antimicrobial stewardship features and microbiology laboratory specifics. For each patient, we collected demographic data and the main diagnosis at ICU admission, including ICU admission for SARS-CoV-2 infection. Comorbidities were assessed using the five markers of the Chronic Health Evaluation of the APACHE II score and the Charlson index [11, 12]. Severity of illness was defined at ICU admission by the Simplified Acute Physiology Score II (SAPS II) [13], and at HABSI diagnosis using the Sequential Organ Failure Assessment (SOFA) score. Data on antimicrobial exposure from one week prior to the study infection were routinely collected. Further information on definitions is illustrated in the electronic supplementary material (ESM).

For each microorganism, we routinely collected: date and time of blood culture sampling, category according to Gram-stain, phenotypical resistance and, when available, genotypical resistance mechanisms. Carbapenem resistance for Enterobacterales was defined according to the U.S. Centers for Diseases Control and Prevention (CDC) as resistant to at least one carbapenem [14]. Difficult-to-treat resistance (DTR) in Gram-negative bacteria was defined as resistance to all first-line antimicrobials (carbapenems, fluoroquinolone, cephalosporins). It was assessed for *Enterobacterales*, *Pseudomonas* spp., and *Acinetobacter* spp., and required all three categories reported plus an assessment of sensitivities to piperacillin-tazobactam or aztreonam if available as outlined by Kadri et al. [15]. Our primary outcome was the distribution of microorganism. Our secondary outcome was mortality. Patients were followed for up to 28 days or until hospital discharge, for further HABSI, duration of organ support, length of ICU and hospital stay, and vital status. Data quality and processes were detailed in the ESM.

### Statistical analyses

Characteristics of centers and patients were described as count (percent) or median (interquartile range) for qualitative and quantitative variables, respectively. Only first episodes of HABSI were analyzed. First, we described the differences between patients using chi-square (or Fisher) and Wilcoxon tests for categorical and numeric variables, respectively. Second, we described the difference in sources of infection and microorganisms’ distribution. In order to mitigate the bias of time-to-HABSI, we performed a sensitivity analysis including only ICU-acquired HABSI, thus excluding patients transferred to the ICU for HABSI management. Third, we performed an explanatory analysis that compared COVID-19 and non-COVID-19 with HABSIs due *only* to enterococci or DTR Gram-negative microorganisms. Fourth, a graphical representation with Kaplan–Meier curves (with log-rank test) using mortality as an outcome was performed. Finally, we tested the association between COVID-19 status and mortality using a multivariable fragility Cox model. A random effect for center was included. For the multivariable analysis, we imputed the solely missing value (i.e., BMI) among the included covariates at the median. Further details on methods were illustrated in the ESM.

All statistical analyses were performed with SAS (version 9.4) and R (Version 3.5.3).

### Ethics

This study was approved by the ethics Committee from the Royal Brisbane & Women's Hospital Human Research (LNR/2019/QRBW/48376); further details were illustrated in the ESM.

## Results

### Centers

**Fig. 1:**
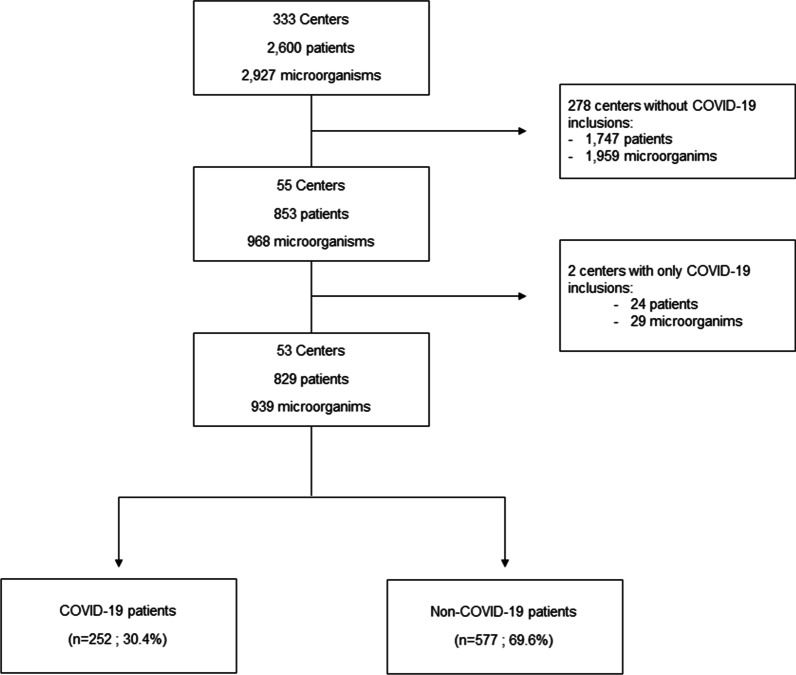
Flowchart

Among the 333 centers recruited in the Eurobact II study, we excluded 278 centers that did not include HABSI in COVID-19 patients (Fig. 1). In addition, two centers included only HABSI in COVID-19 patients and were therefore excluded.

Finally, 53 centers from 19 countries were included (Additional file 1: Fig. S1, Additional file 1: Table S1). Centers were mostly located in Europe and central Asia (*n* = 42, 79%) and were mostly from high-income countries (*n* = 33, 62%). We recruited in median 10 (IQR 7; 20) patients per center, with 25% (IQR 14; 46) of them being COVID-19 patients.

### Patients, HABSIs characteristics and microorganisms

We included 829 patients with a HABSI. Their median age was 65 years old (IQR 55; 74) and 538 (64.9%) were male (Table 1).

**Table 1:** Patients' characteristics on admission, at HABSI time and outcome

Variable	All HABSI (*n* = 829)	COVID-19 patient (*n* = 252)	Non-COVID-19 patient (*n* = 577)	*p value*
Patient characteristics on ICU admission:				
Time from hospital admission to HABSI onset	15 [8; 27]	14 [9; 23]	15 [8; 29]	0.69
Time from ICU admission to HABSI	9 [4; 17]	10 [6; 16]	8 [2; 17]	0.017
Age, years	65 [55; 74]	66 [56; 74]	65 [55; 74]	0.30
Gender				
Female	291 (35.1)	80 (31.7)	211 (36.6)	0.18
Male	538 (64.9)	172 (68.3)	366 (63.4)	
BMI^1^	26.8 [24.1; 30.4]	27.8 [24.9; 31.7]	26.3 [23.4; 29.4]	< 0.0001
Comorbidities				
Respiratory	147 (17.7)	33 (13.1)	114 (19.8)	0.021
Cardio-vascular	191 (23)	57 (22.6)	134 (23.2)	0.85
Neurological	137 (16.5)	27 (10.7)	110 (19.1)	0.0029
Metabolic disorders	326 (39.3)	107 (42.5)	219 (38)	0.22
Gastro-intestinal	49 (5.9)	13 (5.2)	36 (6.2)	0.54
Immunosuppression	122 (14.7)	15 (6)	107 (18.5)	< 0.0001
Malignancy	143 (17.2)	19 (7.5)	124 (21.5)	< 0.0001
Steroids for sepsis or septic shock^2^	204 (25)	74 (29.7)	130 (22.9)	0.038
ICU admission origin				
Emergency department	277 (33.4)	73 (29)	204 (35.4)	< 0.0001
Hospital ward/floor	327 (39.4)	111 (44)	216 (37.4)	
Operating room/recovery	67 (8.1)	1 (0.4)	66 (11.4)	
Other hospital	118 (14.2)	47 (18.7)	71 (12.3)	
Other intermediate care unit	26 (3.1)	10 (4)	16 (2.8)	
Other	14 (1.7)	10 (4)	4 (0.7)	
Admission type				
Medical	693 (83.6)	249 (98.8)	444 (76.9)	< 0.0001
Surgical elective	30 (3.6)	2 (0.8)	28 (4.9)	
Surgical emergency	106 (12.8)	1 (0.4)	105 (18.2)	
SAPS II	47 [37; 58]	42 [33; 50]	49 [38; 62]	< 0.0001
Glasgow coma scale^3^	14 [8; 15]	15 [13; 15]	12 [6; 15]	< 0.0001
Ventilation status				
High-flow oxygen nasal cannula	76 (9.2)	35 (13.9)	41 (7.1)	< 0.0001
Invasive mechanical ventilation	510 (61.5)	147 (58.3)	363 (62.9)	
Low-flow oxygen or no oxygen	153 (18.5)	32 (12.7)	121 (21)	
Non-invasive mechanical ventilation or CPAP	90 (10.9)	38 (15.1)	52 (9)	
Patient characteristics at HABSI diagnosis:				
Adrenaline	37 (4.5)	13 (5.2)	24 (4.2)	0.52
Noradrenaline^4^	413 (49.9)	127 (50.4)	286 (49.7)	0.84
SOFA	8 [5; 11]	8 [4; 11]	8 [5; 12]	0.13
Glasgow coma scale^5^	12 [7; 15]	14 [8; 15]	10 [6; 15]	< 0.0001
Ventilation status				
High-flow oxygen nasal cannula	42 (5.1)	13 (5.2)	29 (5)	0.0003
Invasive mechanical ventilation	617 (74.4)	210 (83.3)	407 (70.5)	
Low-flow oxygen or no oxygen	123 (14.8)	23 (9.1)	100 (17.3)	
Non-invasive mechanical ventilation or CPAP	47 (5.7)	6 (2.4)	41 (7.1)	
Source control required but not achieved	80 (9.7)	20 (7.9)	60 (10.4)	0.27
Adequate empirical therapy within the first 24h^6^	343 (48.5)	96 (48.2)	247 (48.4)	0.96
Corticosteroids for sepsis or septic shock	204 (25%)	74 (29.7)	130 (22.9)	0.038
Status at day 28				
Alive in the Hospital	133 (16)	25 (9.9)	108 (18.7)	< 0.0001
Alive in the ICU	206 (24.8)	55 (21.8)	151 (26.2)	
Death in the Hospital	16 (1.9)	3 (1.2)	13 (2.3)	
Death in the ICU	363 (43.8)	145 (57.5)	218 (37.8)	
Discharged from the Hospital	111 (13.4)	24 (9.5)	87 (15.1)	
28-day mortality	379 (45.7)	148 (58.7)	231 (40)	< 0.0001

*HA-BSI* hospital-acquired bloodstream infection, *ICU* intensive care unit, *SAPS* Simplified Acute Physiology Score, *SOFA* sequential organ failure assessment score, *CPAP* continuous positive airway pressure

Results reported as *n* (%) for categorical variables and median [IQR] for continuous variables. Missing Data (MD): ^1^BMI: 1 MD. ^2^Steroids for sepsis or septic shock: 12 MD. ^3^Glasgow coma scale on admission: 10 MD. ^4^Noradrenalin at HA-BSI onset: 1 MD. ^5^Glasgow coma scale at HA-BSI onset: 7 MD. ^6^Adequate treatment in the first 24 h with 120 MD

The most frequently observed comorbidities were the metabolic ones (*n* = 326, 39.3%), followed by cardio-vascular (*n* = 191, 23%) and respiratory (*n* = 147, 17.7%), and malignancies (*n* = 143, 17.2%). More than 80% of patients were admitted to ICU for a medical reason, with a median SAPS II on admission at 47 (IQR 37; 58); 617 (74.4%) were receiving invasive mechanical ventilation at HABSI onset.

The most frequently observed sources of infection were an intravascular catheter (*n* = 257, 31%) and the respiratory tract (*n* = 251, 30.3%, Table 2).

**Table 2:** Source of infections and microorganism groups

Variable	All HABSI (*n* = 829)	COVID-19 patient (*n* = 252)	Non-COVID-19 patient (*n* = 577)	*p value*
Previous antibiotics:				
Antimicrobials received within the 7 days prior HABSI	622 (75)	204 (81)	418 (72.4)	0.0092
Source of infection:				
Intravascular catheter	257 (31)	74 (29.4)	183 (31.7)	0.50
Respiratory tract	251 (30.3)	101 (40.1)	150 (26)	< 0.0001
Primary HABSI	163 (19.7)	64 (25.4)	99 (17.2)	0.0060
Intra-abdominal tract	75 (9)	3 (1.2)	72 (12.5)	< 0.0001
Bones and soft tissues	43 (5.2)	5 (2)	38 (6.6)	0.0060
Urinary tract	35 (4.2)	8 (3.2)	27 (4.7)	0.32
Other (endocarditis, mediastinitis, central nervous system)	28 (3.4)	4 (1.6)	24 (4.2)	0.059
Multiple first sources of infection	23 (2.8)	7 (2.8)	16 (2.8)	0.997
Microorganism group:				
Gram-positive bacteria	285 (34.4)	100 (39.7)	185 (32.1)	0.033
Resistant* Gram-positive bacteria	108 (13)	32 (12.7)	76 (13.2)	0.85
Gram-negative bacteria	505 (60.9)	151 (59.9)	354 (61.4)	0.70
DTR Gram-negative bacteria	124 (15)	49 (19.4)	75 (13)	0.017
Fungi	79 (9.5)	19 (7.5)	60 (10.4)	0.20
Anaerobic bacteria	16 (1.9)	1 (0.4)	15 (2.6)	0.050
Polymicrobial HABSI	97 (11.7)	37 (14.7)	60 (10.4)	0.078

Results reported as *n* (%) for categorical variables and median [IQR] for continuous variables.

*HABSI* hospital-acquired bloodstream infection, *DTR* difficult-to-treat resistance.

*For example methicillin-resistant *S. aureus*.

We identified 939 microorganisms: *Klebsiella* spp. (*n* = 147, 15.7%), *Acinetobacter* spp (*n* = 143, 15.2%) and enterococci (*n* = 118, 12.6%) were the most frequently detected microorganisms. The rate of DTR Gram-negative microorganisms was 15%. *S. aureus* was identified only in 79 HABSIs (8.4%).

### Differences between COVID-19 and non-COVID-19 patients

We included 252 COVID-19 and 577 non-COVID-19 patients. COVID-19 patients had fewer comorbidities (Table 1). On ICU admission, COVID-19 patients had lower SAPS II scores (median 42 [IQR 33; 50] vs. 49 [IQR 38;62], *p* < 0.0001) and were frequently receiving high-flow oxygen nasal cannula (13.9% vs. 7.1%) and non-invasive mechanical ventilation (15.1% vs. 9%, *p* < 0.0001) compared to non-COVID-19 patients.

The time between hospital admission and HABSI was similar between COVID-19 (14 [IQR 9; 23] days) and non-COVID-19 patients (15 [IQR 8; 29] days, *p* = 0.69). However, ICU-acquired HABSI in COVID-19 patients (10 days, IQR 6; 16) occurred later compared to non-COVID-19 patients (8, [IQR 2; 17], *p* = 0.02). COVID-19 patients were more frequently exposed to antimicrobials in the week before the occurrence of HABSI (81.0% vs. 72.4% in non-COVID-19 patients, *p* = 0.009). Ceftriaxone was most frequently administered in COVID-19 patients (9.0% vs. 6.4% in non-COVID-19 patients, *p* = 0.089, Additional file 1: Table S2). No significant differences in other antimicrobials were observed (Additional file 1: Table S2).

HABSIs in COVID-19 patients were most often from respiratory sources (40.1% vs. 26.0%, *p* < 0.0001) and primary HABSI (25.4% vs. 17.2%, *p* = 0.006), whereas HABSIs in non-COVID-19 patients were most often related to intraabdominal (12.5% vs. 1.2%, *p* < 0.0001) and bone/soft tissues (6.6% vs. 2.0%) infections (Table 2). Gram-positive bacteria were most often involved in COVID-19 patients HABSIs (39.7% vs. 32.1%, *p* = 0.033). Interestingly, HABSI due to DTR Gram-negative were more often observed in COVID-19 patients. Of note, a sensitivity analysis including only ICU-acquired HABSI showed similar sources of infection and microorganism distribution compared to the main analysis between COVID-19 and non-COVID-19 patients (Additional file 1: Tables S3–S4).

**Fig. 2:**
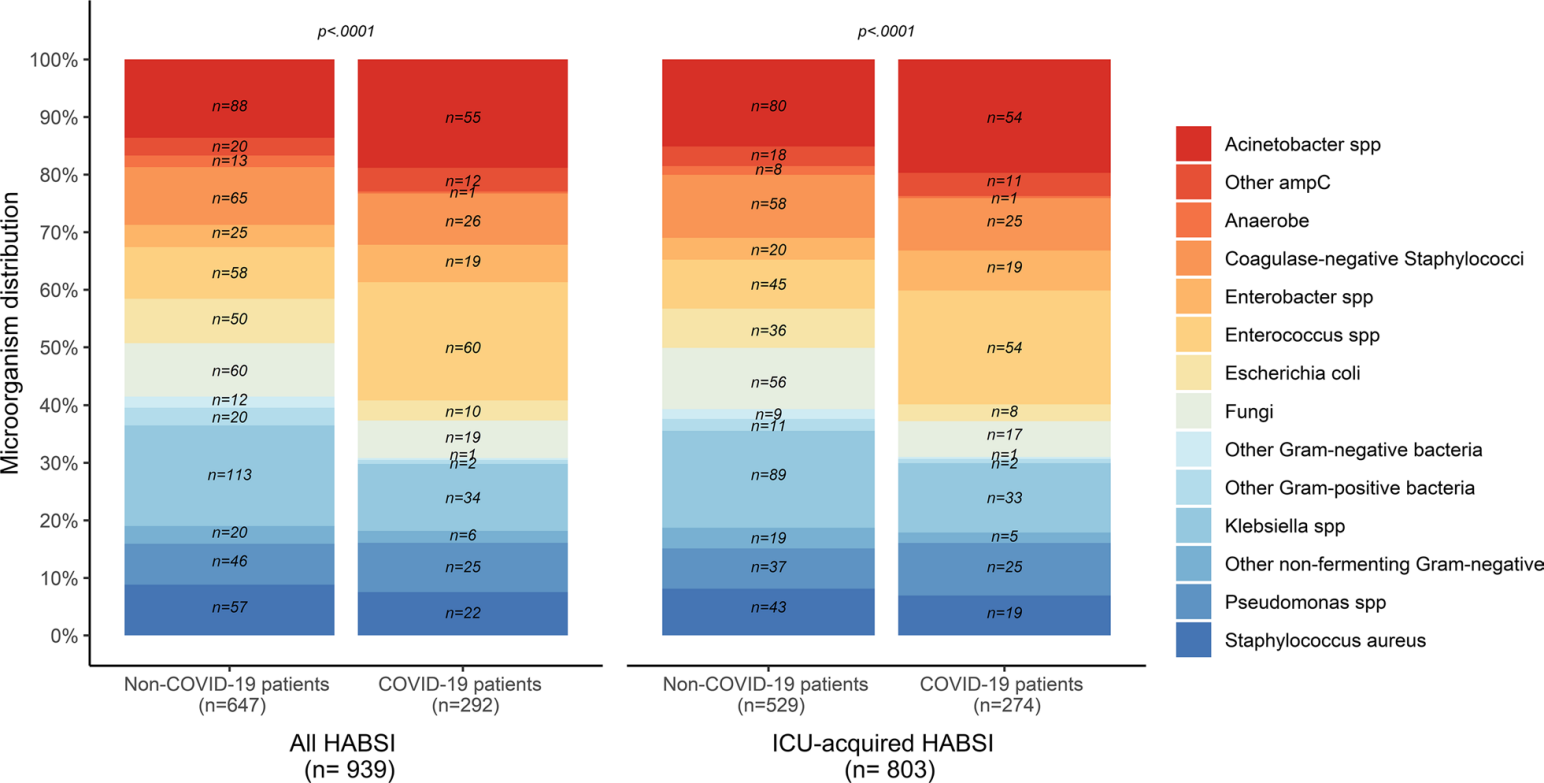
Distribution of microorganisms between COVID-19 and non-COVID-19 patients in all HABSI and in ICU-acquired HABSI. *HA-BSI* hospital-acquired bloodstream infection, *ICU* intensive care unit, *spp.* species

Figure 2 shows the distribution of microorganisms between COVID-19 and non-COVID-19 patients. HABSIs in COVID-19 patients were most frequently caused by enterococci (20.5% vs. 9.0%) and *Acinetobacter* spp. (18.8% vs. 13.6%), whereas those in non-COVID-19 patients were most frequently caused by *Klebsiella* spp. (17.5% vs. 11.6%, *p* < 0.0001). Distribution of microorganism during the ICU length-of-stay is illustrated in Additional file 1: Fig. S2.

### Enterococcal HABSIs

*E. faecalis* accounted for 45% (*n* = 27) and 34.5% (*n* = 20) of enterococcal HABSIs in the COVID-19 and non-COVID-19 group, respectively. *E. faecium* proportion were similar in COVID-19 (*n* = 32, 53.3%) and non-COVID-19 (*n* = 33, 56.9%) patients. Similar proportions of vancomycin-resistant *E. faecium* (VRE) were observed in both groups. COVID-19 patients with enterococcal HABSI were less often immunosuppressed (3.4% vs. 29.3% in non-COVID-19; *p* = 0.0002) and had less often malignancy (5.2% vs. 25.9%, *p* = 0.002, Additional file 1: Table S5). Primary (*n* = 26, 44.8%) enterococcal HABSIs were more frequent in COVID-19 patients compared to non-COVID-19 patients (*n* = 11, 19.0%, *p* = 0.0028). HABSI were frequently assigned to an abdominal source in non-COVID-19 patients (*n* = 13, 22.4%). Polymicrobial enterococcal HABSI were more frequently but non-statistically significantly observed in non-COVID-19 patients (36.2% vs. 25.6%, *p* = 0.23).

### Gram-negative DTR HABSIs

HABSIs due to DTR Gram-negative pathogens occurred a median 11 days (IQR 8;18) after hospital admission in COVID-19 patients, whereas in non-COVID-19 were observed after 20 days (IQR 10; 40, *p* = 0.001, Additional file 1: Table S6).

*Acinetobacter* spp. accounted for 60.3% (*n* = 35) of Gram-negative DTR in COVID-19 patients and *Klebsiella* spp. accounted for 40.2% (*n* = 35) of Gram-negative DTR in non-COVID-19 patients (Additional file 1: Table S7).

### Mortality

**Fig. 3:**
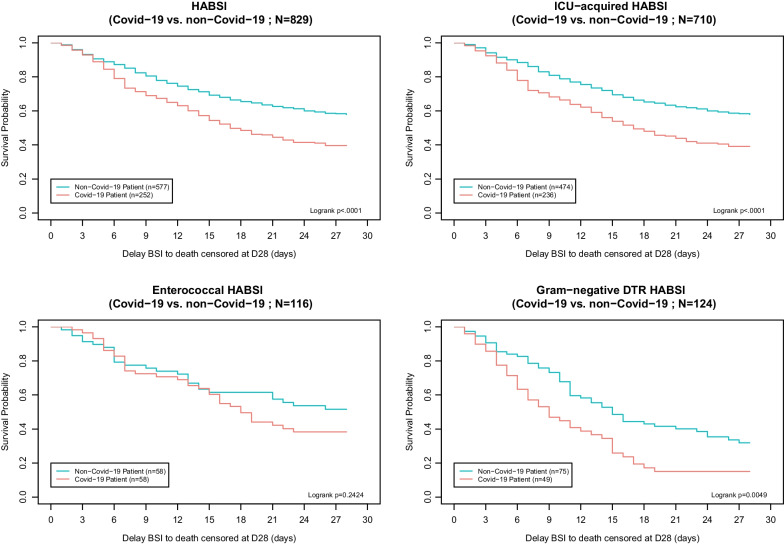
Survival curves for all, ICU-acquired, enterococcal and DTR Gram-negative HABSI. *ICU* intensive care unit, *HA-BSI* hospital-acquired bloodstream infection, *DTR* difficult to treat resistance, *Vs* versus

Overall, the day-28 mortality rate was 45.7% (*n* = 379), reaching 58.7% in COVID-19 patients, versus 40.0% for non-COVID-19 patients (*p* < 0.0001, Fig. 3). In patients with Gram-negative DTR HABSIs, the day-28 mortality was also higher for COVID-19 (83.7% vs. 65.3% in non-COVID-19, *p* = 0.025, Fig. 3).

Using a multivariable fragility Cox model, we observed a significant association between COVID-19 status and mortality (hazard ratio 1.91, 95% CI 1.49–2.45, *p* < 0.0001, Additional file 1: Table S8).

## Discussion

Using a large multicontinental prospective cohort, we showed that the epidemiology of HABSI in critically ill patients was different between COVID-19 and non-COVID-19 patients. Enterococcal HABSIs were more frequently observed in COVID-19 patients.

Several studies showed that enterococcal HABSIs were frequent in critically ill COVID-19 patients, ranging from 25% to almost 50% of HABSI [16, 17]. Interestingly, only few studies have reported the differences between COVID-19 and non-COVID-19 critically ill patients with HABSI. The first study matched critically ill COVID-19 patients with similar non-COVID-19 patients and showed a higher rate of enterococcal HABSI among COVID-19 patients [3]. However, this study (1) included a limited number of patients; (2) reported a relatively small number of all-causes HABSI without enterococcal HABSI in non-COVID-19 patients; and (3) included non-COVID-19 patients prior the COVID-19 pandemic. The second study was a small monocentric retrospective cohort study that compared SARS-CoV-2 or influenza patients with inpatients without positive SARS-CoV-2 or influenza tests during the study period. Enterococci were detected in 6 of 20 bacteremic COVID-19 patients, whereas in critically ill influenza patients no enterococcal HABSI was observed [18]. Both studies, due to the small numbers of patients included, showed only a trend towards an increased proportion of enterococcal HABSI in COVID-19 ICU patients. Using high-quality data from a large multicontinental prospective cohort, we found that enterococcal HABSIs were more frequently observed in critically ill COVID-19 patients. A subgroup analysis including only ICU-acquired HABSI illustrated that the enterococcal frequency was increased in this subpopulation. This finding could have several explanations. First, enterococci colonized the gastrointestinal tract [19]. Even if Eurobact II investigators rarely allocated HABSIs to the abdominal source in COVID-19 patients, it is conceivable that more abdominal translocations could occur in COVID-19 patients and were allocated by our investigators to primary HABSI. Critically ill COVID-19 were at a particularly high risk for developing gastrointestinal complications ranging from acute cholecystitis or pancreatitis to ileus or mesenteric ischemia [20–24]. Indeed, SARS-CoV-2 has been detected in the gastrointestinal tract and it may enter gastrointestinal cells via angiotensin-converting enzyme 2 receptors, which are highly expressed in the gastrointestinal tract, to cause direct damage to gastrointestinal organs [25–29]. The microvascular inflammatory coagulopathy of COVID-19 leading to higher incidence of deep vein thrombosis may be another pathophysiological mechanism possibly leading to bacterial translocation. An inflammatory coagulopathy may be associated with deep vein thrombosis or cerebrovascular accidents [30–32]: it is conceivable that a similar mechanism may lead to mesenteric ischemia and, therefore, may increase the proportion of detected enterococcal BSI. Second, our study suggested that COVID-19 patients were more frequently exposed to antimicrobials. Cephalosporins are often ineffective against enterococcal species and their prior use was demonstrated to be a major risk factor for the acquisition of enterococcal infections [33, 34]. Previous exposure to antibiotics is unlikely to be the sole explanation for our findings but it can be an instrumental concomitant factor leading to increased proportion of enterococcal HABSI in critically ill patients with COVID-19. Third, enterococci, in particular VRE, may be a marker for poor infection prevention and control (IPC) measures and hand hygiene compliance [35]. The COVID-19 pandemic produced many challenges for IPC, including unit over-crowding, fatigue and sessional use of PPE. These factors likely reduced compliance with IPC measures and contributed to a rise in nosocomial infections. In this context, it is possible that intravascular catheters were more frequently contaminated and subsequently infected with enterococci. However, HABSI patients with and without COVID-19 were recruited for this study during the same period and we did not observe a predominance of VRE or a specific enterococcal species (*faecalis* vs. *faecium*) in COVID-19 patients, thus suggesting this was not the dominant cause of the effects seen. Interestingly, a tendency towards more HABSI due to *Acinetobacter* spp. [36] in COVID-19 patients was observed. This result remains intriguing: several outbreaks of *Acinetobacter* spp. were observed during the COVID-19 pandemic and, therefore, a possible impact of reduced IPC measures in the solely COVID-19 population might be hypothesized [37].

Our results have several clinical implications. Whether empirical therapy with glycopeptides or oxazolidinones should be administered in septic in critically ill patients with abdominal sepsis is still debated [38]. A recent multicentric study showed that an initial antibiotic treatment which did not cover enterococci was associated with an increased mortality in critically ill patients with a microbiologically confirmed intra-abdominal infection with *Enterococcus* spp. [39]. In light of these considerations, for septic critically ill COVID-19 patients, an empirical therapy covering all enterococcal species should be considered, especially when a third-generation cephalosporin was previously used. Due to the less pronounced results for resistant Gram-negative microorganisms, no firm conclusions on empirical antibiotic therapy for Gram-negative can be provided.

Our study has several limitations. First, on one hand, the Eurobact II was designed prior to the COVID-19 pandemic. Therefore, several important SARS-CoV-2 variables (e.g., SARS-CoV-2 specific therapies [corticosteroids, tocilizumab] that could affect bacterial infectious risk, SARS-CoV-2 genotype and ICU overcrowding data) were not routinely collected and could not be analyzed. Immunosuppressive drugs administered for COVID-19 may impact on HABSI epidemiology even if large randomized controlled trials did [40, 41] not show a substantial impact on subsequent infections. Moreover, several HABSI patients in our cohort did not receive immunosuppressive drugs according to our definitions, thus highlighting a COVID-19 population during the pre-tocilizumab era. On the other hand, the Eurobact II study, thanks to huge efforts from the local investigators despite the pandemic crisis, allowed an analysis in ICU that prospectively recruited HABSI in both COVID-19 and non-COVID-19 patients, thus mitigating this selection bias. Second, centers recruited patients during different periods, and COVID-19 were not matched with non-COVID-19 patients. Third, four countries (Turkey, France, United Kingdom, and Italy) recruited 50% of patients, thus potentially limiting the generalizability of our results. However, at least one country of all five continents recruited patients for this study. Fourth, centers were allowed to extend the number of inclusions, thus leading to an imbalance of the total number of HABSI recruited between the various centers. Finally, denominator data (i.e., ICU admissions) were not provided by all centers, thus limiting the interpretation of our results.

## Conclusions

Using a large multicontinental prospective cohort, we showed that the epidemiology of HABSI differed between COVID-19 and non-COVID-19 patients, with enterococcal HABSI being disproportionately more common in COVID-19 patients. Despite less comorbidities and lower severity scores on admission, COVID-19 patients with HABSI had significantly higher mortality than patients with HABSI but without COVID-19.

## Supplementary Information

**Additional file 1.** Additional methods (definitions, additional methods, statistical analyses and ethics), Additional tables (Tables S1–S8) and Additional figures (Figs. S1–S2).

## Data Availability

The datasets used and/or analyzed during the current study are available from the corresponding author on reasonable request.

## Abbreviations

BMI: Body Mass Index
BSI: Bloodstream infections
CDC: Centers for Diseases Control and Prevention
CI: Confidence interval
CRF: Case report form
DTR: Difficult-to-treat resistance
ESICM: European Society of Intensive Care Medicine
ESGCIP: Infectious Diseases (ESCMID) study Group for Infections in Critically Ill Patients
ESM: Electronic supplementary material
HABSI: Hospital-acquired bloodstream infection
HR: Hazard ratio
ICU: Intensive Care Unit
IPC: Infection prevention and control
IQR: Interquartile range
NCs: National coordinators
SAPS: Simplified Acute Physiology Score
SOFA: Sequential organ failure assessment
SARS-CoV-2: Severe acute respiratory syndrome coronavirus 2
