# Supplementary material for: Different epidemiology of bloodstream infections in COVID-19 compared to non-COVID-19 critically ill patients: a descriptive analysis of the Eurobact II study
Source: Crit Care. 2022 Oct 18;26:319. doi: 10.1186/s13054-022-04166-y (PMC9578203; doi:10.1186/s13054-022-04166-y)

Additional file - Electronic supplementary material

Title: Different epidemiology of bloodstream infections in COVID-19 compared to non-COVID-19 critically ill patients: a descriptive analysis of the Eurobact II study

Journal: Critical care.

Authors: Buetti N[1,2], Tabah A[3], Loiodice A[4], Ruckly S[4], Aslan AT[5], Montrucchio G[6], Cortegiani A[7], Saltoglu N[8], Kayaaslan B[9], Aksoy F[10], Akova M[11], Akdoğan Ö[12], Saracoglu KT[13], Erdogan C[14], Leone M[15], Ferrer R[16], Paiva JA[17], Hayashi Y[18], Ramanan M[19], Conway Morris A[20], Barbier F[21], Timsit JF[22]

Affiliations:

1) Infection Control Program and WHO Collaborating Centre on Patient Safety, Geneva University Hospitals and Faculty of Medicine, Rue Gabrielle-Perret-Gentil 4, 1205 Geneva, Switzerland.
2) University of Paris, INSERM, IAME, U1137, Team DeSCID, Paris, France.
3) Intensive Care Unit, Redcliffe Hospital, Metro North Hospital and Health services, Queensland, Australia; Queensland University of Technology, Brisbane, Queensland, Australia; Faculty of Medicine, University of Queensland, Brisbane, Queensland, Australia
4) ICURESEARCH, 26 rue Garibaldi, Fontaine
5) Hacettepe University, Department of Internal Medicine, Sihhiye, Ankara, Turkey
6) Department of Anaesthesia, Critical Care and Emergency, Città Della Salute e Della Scienza Hospital, Corso Dogliotti 14, 10126 Turin, Italy.
7) Department of Surgical Oncological and Oral Science (Di.Chir.On.S.), University of Palermo. Department of Anesthesia, Intensive Care and Emergency, Policlinico Paolo Giaccone, Palermo, Italy.
8) Istanbul University-Cerrahpasa, Department of Infectious Diseases and Clinical Microbiology
9) Ankara Yıldırım Beyazıt University, Ankara City Hospital, Department of Infectious Diseases and Clinical Microbiology
10) Karadeniz Technical University, Department of Infectious Diseases and Clinical Microbiology
11) Hacettepe University, Department of Infectious Diseases and Clinical Microbiology
12) Hitit University, Erol Olçok Research and Training Hospital, Department of Infectious Diseases and Clinical Microbiology
13) Kartal Dr. Lütfi Kırdar City Hospital, Department of Anesthesiology and Reanimation

14) Medipol Mega Hospital, Department of Anesthesiology and Reanimation
15) Department of Anesthesiology and Intensive Care Unit, Hospital Nord, Aix Marseille University, Assistance Publique Hôpitaux Universitaires de Marseille, Marseille, France.
16) Intensive Care Department, Hospital Universitari Vall d'Hebron. SODIR Research Group Vall d'Hebron Institut de Recerca, Barcelona, Spain.
17) Intensive Care Medicine Department. Centro Hospitalar Universitário São João (CHUSJ). Porto. Department of Medicine. Faculty of Medicine. University of Porto (FMUP). Porto. Portugal.
18) Department of Intensive Care Medicine, Kameda Medical Center, Kamogawa, Japan.
19) Caboolture and The Prince Charles Hospitals, Metro North Hospital and Health Services, Brisbane, Queensland, Australia; Critical Care Division, The George Institute for Global Health, University of New South Wales, Sydney, Australia; School of Medicine, University of Queensland, St Lucia, Australia
20) Division of Anaesthesia, Department of Medicine, University of Cambridge, Cambridge, United Kingdom. Division of Immunology, Department of Pathology, University of Cambridge, Cambridge, United Kingdom. JVF Intensive Care Unit, Addenbrooke’s Hospital, Cambridge, United Kingdom

21) Médecine Intensive Réanimation, Centre Hospitalier Régional d'Orléans, Orléans, France. Centre d'Étude des Pathologies Respiratoires (CEPR), INSERM U1100, Université de Tours, Tours, France
22) Medical and Infectious Diseases Intensive Care Unit, AP-HP, Bichat-Claude Bernard University Hospital, 46 rue Henri Huchard, 75877, Paris Cedex, France.

Supplementary material

Definitions

- Intensive care unit: ICUs eligible to participate were defined as managing patients with organ failures within a health-care facility and able to provide invasive mechanical ventilation for a duration of at least 24 hours.
- Admission source: refers to where was the patient prior to admission to the ICU.
- Primary diagnosis: The main reason for admission to the ICU. Only one primary diagnosis should be entered (see codes). If surgical admission the site of surgery should be entered as primary diagnosis.
- Type of admission: Surgical - defined as having surgery within 7 days of ICU admission. Elective surgery was defined as surgery scheduled > 24 hours in advance and emergency surgery as that scheduled within 24 hours of operation. All other admissions were considered medical.
- HA-BSIs were defined as isolation of a pathogenic organism from at least one blood culture 48 hours or more after hospital admission; the same 48-hour criterion was used to define ICU-acquired cases among HA-BSIs. For common skin contaminants (coagulase-negative staphylococci, *Corynebacterium* species, *Bacillus* species, *Propionibacterium* species, *Aerococcus* species, *Micrococcus* species), two blood cultures with the same antimicrobial susceptibility profile were mandatory or strong clinical grounds that it is not a contaminant. One example was infected material proven as a source for the HA-BSI. Where strong evidence supported HA-BSI but only one culture was positive (*e.g.,* positive catheter tip following line removal for suspected infection with prescription of additional treatment), all clinical and microbiological data were reviewed to decide whether the case should be included. Patients with BSIs acquired outside the ICU were eligible for inclusion if less than 2 days elapsed between collection of the first positive blood sample and ICU admission and/or if ICU admission was directly related to the consequences of HA-BSI. The inclusion date was the time of collection of the first positive blood culture.
- Comorbidities: Chronic diseases present prior to ICU admission. More than one can be chosen according to the following definitions:
- Metastatic cancer: Metastases proven by surgery, computed tomography or magnetic resonance scan, or any other method.
- Hematologic cancer: Lymphoma, Leukaemia.
- AIDS: HIV positive patients with clinical complications such as Pneumocystis carinii pneumonia, Kaposi's sarcoma, lymphoma, tuberculosis, or toxoplasma infection.

- Chronic renal failure: Defined as either chronic dialysis dependent renal failure or history of chronic renal insufficiency with a serum creatinine > 3.6 g/dL (300 µmol/L).
- Immunosuppression: Administration within the 6 months prior to ICU admission of corticosteroid treatment (at least 0.3 mg/kg/day prednisolone for at least one month) or other immunosuppressant drugs, severe malnutrition, congenital immune-humoral or cellular immune deficiency state.
- Chemotherapy/radiotherapy: If within 6 months prior to ICU admission.
- COPD / Chronic Pulmonary Disease Severe: Chronic restrictive, obstructive or vascular disease resulting in severe exercise limitation (*e.g.*, unable to climb stairs or perform household duties) or documented chronic hypoxia, hypercapnia, secondary polycythaemia, severe pulmonary hypertension (>40 mmHg) or home oxygen or non-invasive ventilation (NIV).
- Liver disease, severe: Biopsy-proven cirrhosis with portal hypertension; episodes of past upper gastro-intestinal bleeding attributed to portal hypertension; or prior episodes of hepatic failure, encephalopathy, or coma.
- Metabolic disease: Diabetes with or without end organ damage, moderate renal disease or renal disease receiving chronic dialysis, or connective tissue disease.
- For scoring purposes, we recorded minimal and maximal or worse biological and physiological values of the first 24 hours following ICU admission.
- For the Glasgow coma scale (GCS) we defined the following: For non-sedated patients, enter the lowest GCS during the 24 hours. For patients sedated, enter the GCS at the time of/just prior to sedation. If not available, please enter an estimated GCS score as it would be if the patient was not receiving sedation.
- Decision to withhold or withdraw life-sustaining treatment was defined as the ethical decision to change goal of treatment from life-prolonging to palliative. It should only be entered if organ supportive therapy was stopped or not started when it would otherwise have been indicated
- Methicillin-resistant *Staphylococcus aureus* (MRSA) and methicillin-resistant (coagulase-negative) *Staphylococcus epidermidis* (MRSE) were defined as resistance to methicillin/oxacillin.
- Vancomycin-resistant enterococci (VRE) was defined as resistance to vancomycin.
- Carbapenem resistance for *Enterobacterales* was defined as resistance to at least one carbapenem as recommended by the United States of America Center For Disease Control And Prevention.
- Adequate antimicrobial therapy was defined as the administration of an antibiotic to which the organism was fully susceptible *in vitro*. In the patients without susceptibility

data or with incomplete antibiograms, antimicrobial therapy was considered adequate if the intrinsic organism characteristics and usual susceptibilities indicated a high likelihood of drug susceptibility.

- Time to adequate antimicrobial therapy was defined as the time between sampling of the study blood culture and receipt of one adequate antimicrobial for each pathogen in the blood culture

Sources of Hospital acquired blood stream-infection and source control

The presumed source of the BSI was determined by the treating clinician from the following pre-defined list of categories and subcategories, and if multiple possible sources we requested ordering/numbering in order of likelihood.

- Primary: defined as no clear focus or portal of entry identified
- Catheter-related (Intra-vascular catheter only)
- Respiratory tract
  - Pneumonia
  - Pleural, empyema
  - Tracheobronchitis
- Intra-abdominal
  - Peritonitis
  - Biliary source
  - Other intra-abdominal
- Urinary tract
- Bone or soft tissues
  - Necrotizing fasciitis
  - Other soft tissue
  - Joint or bone
  - Spine
- Endocarditis
- Mediastinitis
- Central Nervous System

Source control was recorded according to the clinician's report as

- Not required
- Required, completed
- Source control required but not achieved

When it was required, we recorded the time, date and effectiveness of the intervention according to pre-defined categories, and if a specimen was sent for microbiology and if it was positive. When patients had multiple interventions we recorded the number of interventions, date of the last intervention and if it was deemed effective after the last intervention.

Source control interventions were recorded according to the following categories

Intravascular catheter Related
- Catheter removal
- Surgical vascular procedure (ligature)

Respiratory tract (pulmonary, pleural, empyema)
- Surgical thoracic

- Percutaneous thoracic (including chest drain)
- Percutaneous mediastinal

Vascular

- Surgical vascular
- Percutaneous vascular
- Other vascular

Cardiac and mediastinal

- Surgical cardiac
- Surgical mediastinal
- Percutaneous mediastinal
- other cardiac or mediastinal

Intra-abdominal

- Surgical abdominal
- Percutaneous abdominal
- Surgical other (mediastinal, pleural, …)
- Percutaneous other (mediastinal, pleural, …)

Urinary tract

- Surgical urinary (JJ stent)
- Surgical urinary (nephrectomy or other)
- Percutaneous urinary (nephrostomy)
- Other urinary

Bone or soft tissues

- Surgical skin
- Surgical bone
- Other bone or soft tissue

Other

- Percutaneous other
- Surgical other

Other

Supplementary methods: data quality and processes

We used a dual verification and query process that included automatic verification of all collected data through a set of coherence routines, and manual checking of each CRF by a group of experts (AT, NB and FB) for data quality, logic, and completeness. Any question or incoherence was fed back to the center's investigator and checked again until satisfactory completion of the case. In the absence of a response, we attempted to contact at least 3 times the investigator and any other available contact in this center. Administered antibiotics were correlated with provided antibiograms by the operational committee to manually code antibiotic adequacy. Complex cases were reviewed at periodic meetings between experts (AT, FB and NB) to resolve any disagreement.

Statistical analysis and ethics

No imputation was performed on missing data for descriptive analyses.

Cox models were adjusted for the following clinically relevant covariates: time from ICU admission to HABSI, comorbidities (*i.e.*, respiratory tract, neurological, immunosuppression, malignancy), SAPS II on ICU admission, SOFA at HABSI onset and source of infection (respiratory or primary HABSI).

This study was approved by the ethics Committee from the Royal Brisbane & Women's Hospital Human Research (LNR/2019/QRBW/48376). Moreover, we required each study center to check the regulatory framework for observational research in their own jurisdiction and to provide written documentation of the authorization to conduct the study in their setting. Each study site investigator was responsible for ethical approval in collaboration with national coordinators.

eTable 1: Centers characteristics

Variable		Centers (n=53)
Country	Argentina	1 (1.9)
	Australia	2 (3.8)
	Belgium	2 (3.8)
	France	7 (13.2)
	India	1 (1.9)
	Iran	2 (3.8)
	Italy	4 (7.5)
	Japan	2 (3.8)
	Mexico	1 (1.9)
	Poland	1 (1.9)
	Portugal	3 (5.7)
	Russian Federation	1 (1.9)
	Saudi Arabia	1 (1.9)
	Spain	5 (9.4)
	Sudan	1 (1.9)
	Switzerland	1 (1.9)
	Turkey	12 (22.6)
	UK	5 (9.4)
	Bosnia and Herzegovina	1 (1.9)
Region	East-Asia and Pacific	4 (7.5)
	Europe and Central Asia	42 (79.2)
	Latin America and the Caribbean	2 (3.8)
	Middle-East and North-Africa	3 (5.7)
	South-Asia	1 (1.9)
	Sub-Saharan Africa	1 (1.9)
Type of Hospital	Non-teaching Hospital	11 (20.8)
	Teaching Hospital	42 (79.2)
Type of ICU	Mixed (medical-surgical)	44 (83)
	Medical	8 (15.1)
	Surgical	1 (1.9)
Structure of ICU	Closed ICU	43 (81.1)
	Open ICU	10 (18.9)
Funding	Public	45 (84.9)
	Private	5 (9.4)
	Mixed	3 (5.7)
Number of Ventilator-equivalent beds in the ICU		14 [11 ; 20]
Number of nurses available in the ICU for patient care at a given time		8 [5 ; 14]
Number of doctors available in the ICU for patient care at a given time		6 [4 ; 9]
Number of doctors at senior level		3 [2 ; 5]
Number of junior or in training doctors		3 [2 ; 5]
Number of patients per center		10 [7 ; 20]
Number of COVID-19 patients per center		2 [1 ; 5]
Proportion of COVID-19 patient per center (%)		25 [14.3 ; 45.5]

Legend. ICU: Intensive care unit. UK: United Kingdom.

Footnotes: Results reported as n (%) for categorical variables and median [IQR] for continuous variables.

eTable 2: Antibiotics before HABSI in COVID-19 and non-COVID-19 patients

Variable	Treatments of all ICU acquired HABSI (n=1337)	Treatments of COVID-19 patient (n=412)	Treatments of Non-COVID-19 patient (n=925)	*p-value*
Amoxicillin and clavulanic acid	41 (3.1)	8 (1.9)	33 (3.6)	0.1114
Carbapenems	209 (15.6)	72 (17.5)	137 (14.8)	0.2154
Cefalotin	6 (0.4)	0 (0)	6 (0.6)	0.1858
Cefazolin	17 (1.3)	4 (1)	13 (1.4)	0.5126
Cefepime	29 (2.2)	9 (2.2)	20 (2.2)	0.9794
Cefixime	2 (0.1)	0 (0)	2 (0.2)	1
Ceftazidime	17 (1.3)	6 (1.5)	11 (1.2)	0.6873
Ceftriaxone	96 (7.2)	37 (9)	59 (6.4)	0.0888
Cefuroxime	2 (0.1)	0 (0)	2 (0.2)	1
Daptomycin	14 (1)	4 (1)	10 (1.1)	1
Fluorochinolons	53 (4)	17 (4.1)	36 (3.9)	0.8393
Linezolid	37 (2.8)	8 (1.9)	29 (3.1)	0.2193
Piperacillin and Tazobactam	186 (13.9)	57 (13.8)	129 (13.9)	0.9568
Sulfamethoxazole and Trimethoprim	17 (1.3)	3 (0.7)	14 (1.5)	0.2366
Teicoplanin	60 (4.5)	17 (4.1)	43 (4.6)	0.6701
Vancomycin	83 (6.2)	29 (7)	54 (5.8)	0.4007

Legend. HABSI: Hospital-acquired bloodstream infection.

Footnotes: Results reported as n (%).

eTable 3: Sources of infection and microorganisms group in patients with ICU-acquired HABSI

Variable	all ICU acquired HABSI (n=710)	COVID-19 patient (n=236)	Non-COVID-19 patient (n=474)	*p-value*
**Source of infection:**				
Intravascular catheter	229 (32.3)	69 (29.2)	160 (33.8)	0.23
Respiratory tract	217 (30.6)	95 (40.3)	122 (25.7)	<0.0001
Primary	147 (20.7)	62 (26.3)	85 (17.9)	0.0098
Intra-abdominal tract	57 (8)	2 (0.8)	55 (11.6)	<0.0001
Bones and soft tissues	29 (4.1)	4 (1.7)	25 (5.3)	0.02
Urinary tract	28 (3.9)	8 (3.4)	20 (4.2)	0.59
Other (endocarditis, mediastinitis, central nervous system)	23 (3.2)	3 (1.3)	20 (4.2)	0.037
Multiple first sources of infection	20 (2.8)	7 (3)	13 (2.7)	0.87
**Microorganism group:**				
Gram-positive bacteria	237 (33.4)	91 (38.6)	146 (30.8)	0.039
Resistant Gram-positive bacteria	92 (13)	30 (12.7)	62 (13.1)	0.89
Gram-negative bacteria	437 (61.5)	145 (61.4)	292 (61.6)	0.97
DTR Gram-negative bacteria	114 (16.1)	48 (20.3)	66 (13.9)	0.028
Fungi	73 (10.3)	17 (7.2)	56 (11.8)	0.057
Anaerobic bacteria	10 (1.4)	1 (0.4)	9 (1.9)	0.1777
Number of microorganisms per patient	1 [1 ; 1]	1 [1 ; 1]	1 [1 ; 1]	0.059
Polymicrobial HABSI	82 (11.5)	35 (14.8)	47 (9.9)	0.054

Legend. HABSI: Hospital-acquired bloodstream infection. DTR: Difficult-to-treat resistance.

Footnotes: Results reported as n (%).

eTable 4: Microorganisms observed in patients with ICU-acquired HABSI

**Variable**	**Items**	**All ICU acquired HABSI microorganisms (n=803)**	**Microorganisms COVID-19 (n=274)**	**Microorganisms non-COVID-19 (n=529)**	***p-value***
Microorganism	*Acinetobacter* spp.	134 (16.7)	54 (19.7)	80 (15.1)	<0.0001
	Anaerobes	9 (1.1)	1 (0.4)	8 (1.5)	
	Coagulase-negative. staphylococci	83 (10.3)	25 (9.1)	58 (11)	
	Enterobacter spp.	39 (4.9)	19 (6.9)	20 (3.8)	
	Enterococcus spp.	99 (12.3)	54 (19.7)	45 (8.5)	
	Escherichia coli	44 (5.5)	8 (2.9)	36 (6.8)	
	Fungi	73 (9.1)	17 (6.2)	56 (10.6)	
	Other Gram-positive bacteria	13 (1.6)	2 (0.7)	11 (2.1)	
	Other Gram-negative bacteria	10 (1.2)	1 (0.4)	9 (1.7)	
	Klebsiella spp.	122 (15.2)	33 (12)	89 (16.8)	
	Other non-fermenting Gram-negative bacilli	24 (3)	5 (1.8)	19 (3.6)	
	Pseudomonas spp.	62 (7.7)	25 (9.1)	37 (7)	
	Staphylococcus aureus	62 (7.7)	19 (6.9)	43 (8.1)	
	Other ampC microorganisms	29 (3.6)	11 (4)	18 (3.4)	

Legend. HABSI: Hospital-acquired bloodstream infection; spp.: species.

Footnotes: Results reported as n (%).

eTable 5: Enterococcal HABSI in COVID-19 and non-COVID-19 patients

Variable		All enterococcal HABSI (n=116)	COVID-19 patient (n=58)	Non-COVID-19 patient (n=58)	*p-value*
**Patient characteristics on ICU admission:**					
Time from hospital admission to HABSI onset		17.5 [10 ; 30]	16 [9 ; 23]	21 [12 ; 33]	0.12
Time from ICU admission to HABSI		8 [2 ; 17.5]	9.5 [4 ; 15]	7.5 [1 ; 23]	0.49
Age, years		66 [55.5 ; 75.5]	69.5 [57 ; 78]	63.5 [54 ; 75]	0.056
Gender	Female	49 (42.2)	23 (39.7)	26 (44.8)	0.57
	Male	67 (57.8)	35 (60.3)	32 (55.2)	
BMI		27.8 [24.4 ; 32]	28.9 [25.7 ; 33.5]	27.2 [22.9 ; 31.3]	0.035
Comorbidities					
Respiratory		17 (14.7)	7 (12.1)	10 (17.2)	0.43
Cardio-Vascular		28 (24.1)	13 (22.4)	15 (25.9)	0.66
Neurological		16 (13.8)	9 (15.5)	7 (12.1)	0.59
Metabolic disorders		45 (38.8)	23 (39.7)	22 (37.9)	0.85
Gastro-intestinal		13 (11.2)	4 (6.9)	9 (15.5)	0.14
Immunosuppression		19 (16.4)	2 (3.4)	17 (29.3)	0.0002
Malignancy		18 (15.5)	3 (5.2)	15 (25.9)	0.0021
Steroids for sepsis or septic shock [1]		25 (22.1)	14 (25)	11 (19.3)	0.47
ICU Admission origin	Emergency department	28 (24.1)	10 (17.2)	18 (31)	<0.0001
	Hospital ward/floor	58 (50)	30 (51.7)	28 (48.3)	
	Operating Room/recovery	9 (7.8)	0 (0)	9 (15.5)	
	Other hospital	14 (12.1)	12 (20.7)	2 (3.4)	
	Other intermediate care unit	4 (3.4)	3 (5.2)	1 (1.7)	
	Other	3 (2.6)	3 (5.2)	0 (0)	
Adrenaline		3 (2.6)	0 (0)	3 (5.2)	0.24
Noradrenaline [2]		51 (44.3)	23 (39.7)	28 (49.1)	0.31
SAPS II		47 [36 ; 57]	42 [33 ; 53]	52.5 [39 ; 64]	0.0046
Glasgow coma scale [3]		15 [11 ; 15]	15 [14 ; 15]	14 [8 ; 15]	0.0012
Ventilation status	High-Flow Oxygen Nasal Canula	9 (7.8)	4 (6.9)	5 (8.6)	0.56
	Invasive Mechanical Ventilation	74 (63.8)	39 (67.2)	35 (60.3)	
	Low-flow Oxygen or no oxygen	17 (14.7)	6 (10.3)	11 (19)	
	Non-Invasive Mechanical Ventilation or CPAP	16 (13.8)	9 (15.5)	7 (12.1)	
**Patient characteristics at HA-BSI diagnosis:**					
Adrenaline		4 (3.4)	1 (1.7)	3 (5.2)	0.62
Noradrenaline [4]		59 (51.3)	31 (53.4)	28 (49.1)	0.64
SOFA		8 [5 ; 12]	7 [5 ; 12]	8 [6 ; 12]	0.094
Glasgow coma scale [5]		13 [9 ; 15]	14 [10 ; 15]	12 [8 ; 15]	0.025
Ventilation status	High-Flow Oxygen Nasal Canula	6 (5.2)	1 (1.7)	5 (8.6)	0.071
	Invasive Mechanical Ventilation	91 (78.4)	51 (87.9)	40 (69)	
	Low-flow Oxygen or no oxygen	13 (11.2)	5 (8.6)	8 (13.8)	
	Non-Invasive Mechanical Ventilation or CPAP	6 (5.2)	1 (1.7)	5 (8.6)	
Antimicrobials received within the 7 days prior to the HABSI		98 (84.5)	51 (87.9)	47 (81)	0.31
**HABSI source:**					
Catheter-related		39 (33.6)	20 (34.5)	19 (32.8)	0.84
Respiratory		15 (12.9)	6 (10.3)	9 (15.5)	0.41
Primary		37 (31.9)	26 (44.8)	11 (19)	0.0028
Intra-abdominal		16 (13.8)	3 (5.2)	13 (22.4)	0.0071
Bones and soft tissues		6 (5.2)	2 (3.4)	4 (6.9)	0.68
Urinary		3 (2.6)	2 (3.4)	1 (1.7)	1
Other (Endocarditis, Mediastinitis, Central Nervous System)		5 (4.3)	0 (0)	5 (8.6)	0.057
**Status at day 28:**					
Status	Alive in the Hospital	17 (14.7)	5 (8.6)	12 (20.7)	0.20
	Alive in the ICU	25 (21.6)	13 (22.4)	12 (20.7)	
	Death in the ICU	4 (3.4)	1 (1.7)	3 (5.2)	
	Discharged from the Hospital	58 (50)	34 (58.6)	24 (41.4)	
28-day mortality		62 (53.4)	35 (60.3)	27 (46.6)	0.14
Polymicrobial HA-BSI		36 (31)	15 (25.9)	21 (36.2)	0.23

Legend. HABSI: Hospital-acquired bloodstream infection. ICU: Intensive care unit. SAPS: Simplified Acute Physiology Score. SOFA: Sequential organ failure assessment score. CPAP: Continuous positive airway pressure.

Footnotes: Results reported as n (%) for categorical variables and median [IQR] for continuous variables. Missing data (MD): [1]Steroids for sepsis or septic shock: 3 MD. [2]Noradrenaline at admission: 1 MD. [3]Glasgow coma scale at admission (1 MD). [4]Noradrenaline at HABSI: 1 MD. [5]Glasgow coma scale at HA-BSI: 1 MD.

eTable 6: DTR Gram-negative HABSI in COVID-19 and non-COVID-19 patients

Variable		All DTR Gram-negative HABSI (n=124)	COVID-19 patient (n=49)	Non-COVID-19 patient (n=75)	*p-value*
**Patient characteristics on ICU admission:**					
Time from hospital admission to HABSI onset		15 [9 ; 30.5]	11 [8 ; 18]	20 [10 ; 40]	0.0011
Time from ICU admission to HABSI		10 [5 ; 18]	8 [6 ; 13]	11 [4 ; 23]	0.39
Age, years		67.5 [57 ; 79]	68 [57 ; 79]	67 [57 ; 79]	0.52
Gender	Female	43 (34.7)	14 (28.6)	29 (38.7)	0.25
	Male	81 (65.3)	35 (71.4)	46 (61.3)	
BMI		25.7 [23.1 ; 28.7]	26.8 [24.6 ; 29.3]	25 [22.5 ; 27.3]	0.0076
Comorbidities					
Respiratory		22 (17.7)	5 (10.2)	17 (22.7)	0.076
Cardio-vascular		41 (33.1)	16 (32.7)	25 (33.3)	0.94
Neurological		21 (16.9)	5 (10.2)	16 (21.3)	0.11
Metabolic disorders		54 (43.5)	21 (42.9)	33 (44)	0.90
Gastro-intestinal		4 (3.2)	1 (2)	3 (4)	1
Immunosuppression		13 (10.5)	2 (4.1)	11 (14.7)	0.060
Malignancy		20 (16.1)	5 (10.2)	15 (20)	0.15
Steroids for sepsis or septic shock [1]		41 (33.3)	21 (42.9)	20 (27)	0.068
ICU Admission origin	Emergency department	48 (38.7)	18 (36.7)	30 (40)	0.46
	Hospital ward/floor	46 (37.1)	21 (42.9)	25 (33.3)	
	Operating Room/recovery	3 (2.4)	0 (0)	3 (4)	
	Other hospital	21 (16.9)	9 (18.4)	12 (16)	
	Other intermediate care unit	6 (4.8)	1 (2)	5 (6.7)	
SAPS II		46 [38 ; 59.5]	44 [39 ; 52]	49 [37 ; 65]	0.057
Glasgow coma scale [2]		13 [8 ; 15]	15 [11 ; 15]	11 [7 ; 14]	<0.0001
Ventilation status	High-Flow Oxygen Nasal Canula	8 (6.5)	5 (10.2)	3 (4)	0.0024
	Invasive Mechanical Ventilation	61 (49.2)	19 (38.8)	42 (56)	
	Low-flow Oxygen or no oxygen	28 (22.6)	7 (14.3)	21 (28)	
	Non-Invasive Mechanical Ventilation or CPAP	27 (21.8)	18 (36.7)	9 (12)	
**Patient characteristics at H-BSI diagnosis:**					
Adrenaline		5 (4)	1 (2)	4 (5.3)	0.65
Noradrenaline		54 (43.5)	23 (46.9)	31 (41.3)	0.54
SOFA		9 [6 ; 12]	8 [7 ; 11]	10 [6 ; 13]	0.22
Glasgow coma scale [3]		9 [5 ; 15]	15 [6 ; 15]	8 [5 ; 12]	0.012
Ventilation status	High-Flow Oxygen Nasal Canula	5 (4)	3 (6.1)	2 (2.7)	0.41
	Invasive Mechanical Ventilation	101 (81.5)	42 (85.7)	59 (78.7)	
	Low-flow Oxygen or no oxygen	13 (10.5)	3 (6.1)	10 (13.3)	
	Non-Invasive Mechanical Ventilation or CPAP	5 (4)	1 (2)	4 (5.3)	
**Status at day 28:**					
Status	Alive in the Hospital	2 (1.6)	0 (0)	2 (2.7)	0.092
	Alive in the ICU	23 (18.5)	7 (14.3)	16 (21.3)	
	Death in the ICU	90 (72.6)	41 (83.7)	49 (65.3)	
	Discharged from the Hospital	9 (7.3)	1 (2)	8 (10.7)	
28-day mortality		90 (72.6)	41 (83.7)	49 (65.3)	0.025
Polymicrobial HABSI		17 (13.7)	8 (16.3)	9 (12)	0.49

Legend. HABSI: Hospital-acquired bloodstream infection. ICU: Intensive care unit. SAPS: Simplified Acute Physiology Score. SOFA: Sequential organ failure assessment score. CPAP: Continuous positive airway pressure.

Footnotes: Results reported as n (%) for categorical variables and median [IQR] for continuous variables. Missing Data (MD): [1]Steroids for sepsis or septic shock: 1 MD. [2]Glasgow coma scale at admission (4 MD). [3]Glasgow coma scale at HABSI: 3 MD.

eTable 7: Distribution of microorganisms among Gram-negative DTR HA-BSI

Variable	All DTR Gram-negative HABSI microorganisms (n=145)	Microorganisms in COVID-19 (n=58)	Microorganisms in non-COVID-19 (n=87)	*p-value*
Acinetobacter spp.	71 (49)	35 (60.3)	36 (41.4)	0.10
Klebsiella spp.	46 (31.7)	11 (19)	35 (40.2)	
Pseudomonas spp.	7 (4.8)	5 (8.6)	2 (2.3)	
Enterobacter spp.	3 (2.1)	1 (1.7)	2 (2.3)	
Escherichia coli	2 (1.4)	0 (0)	2 (2.3)	
Other non-fermenting Gram-negative bacilli	1 (0.7)	0 (0)	1 (1.1)	
Other ampC microorganisms	3 (2.1)	1 (1.7)	2 (2.3)	
Coagulase-negative staphylococci*	5 (3.4)	2 (3.4)	3 (3.4)	
*Staphylococcus aureus**	2 (1.4)	1 (1.7)	1 (1.1)	
Enterococcus spp.*	4 (2.8)	2 (3.4)	2 (2.3)	
Fungi*	1 (0.7)	0 (0)	1 (1.1)	

Legend. *Some Gram-negative DTR HABSI were polymicrobial. DTR: Difficult-to-treat resistance. HABSI: Hospital-acquired bloodstream infection; spp.: species.

Footnotes: Results reported as n (%).

eTable 8: Multivariable fragility Cox model to assess the association between COVID-19 and mortality

	HR [IC 95%]	p-value
**COVID-19**	**1.91 [1.49 ; 2.45]**	**<0.0001**
Time from ICU admission to HABSI	0.99 [0.978 ; 0.994]	0.0007
BMI	0.995 [0.978 ; 1.012]	0.58
Comorbidities		
- Respiratory tract	0.97 [0.74 ; 1.28]	0.84
- Neurological	0.98 [0.72 ; 1.34]	0.91
- Immunosuppression	1.048 [0.75 ; 1.47]	0.78
- Malignancy	1.21 [0.88 ; 1.66]	0.24
SAPS II on ICU admission	1.007 [1.00 ; 1.015]	0.062
SOFA score at HABSI onset	1.15 [1.12 ; 1.18]	<0.0001
Respiratory source	1.32 [1.02 ; 1.70]	0.037
Primary HABSI	1.61 [1.21 ; 2.14]	0.0012

Legend. ICU: Intensive care unit. BMI: Body Mass Index. HA-BSI: Hospital-acquired Bloodstream infection. SAPS: Simplified Acute Physiology Score. SOFA: Sequential organ failure assessment score. Covariates associated with COVID-19 status were selected as adjustment covariates. Co-linearity was checked.

eFigure 1: Centers included

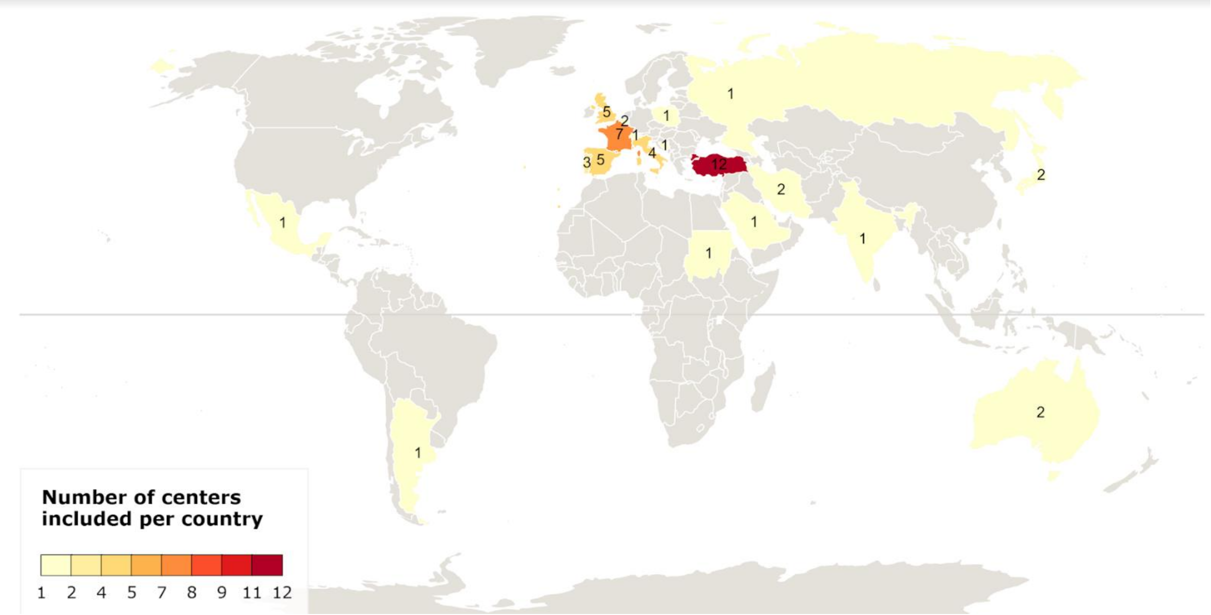

eFigure 2: Distribution of microorganism in COVID-19 and non-COVID-19 HABSI during the ICU length of stay.

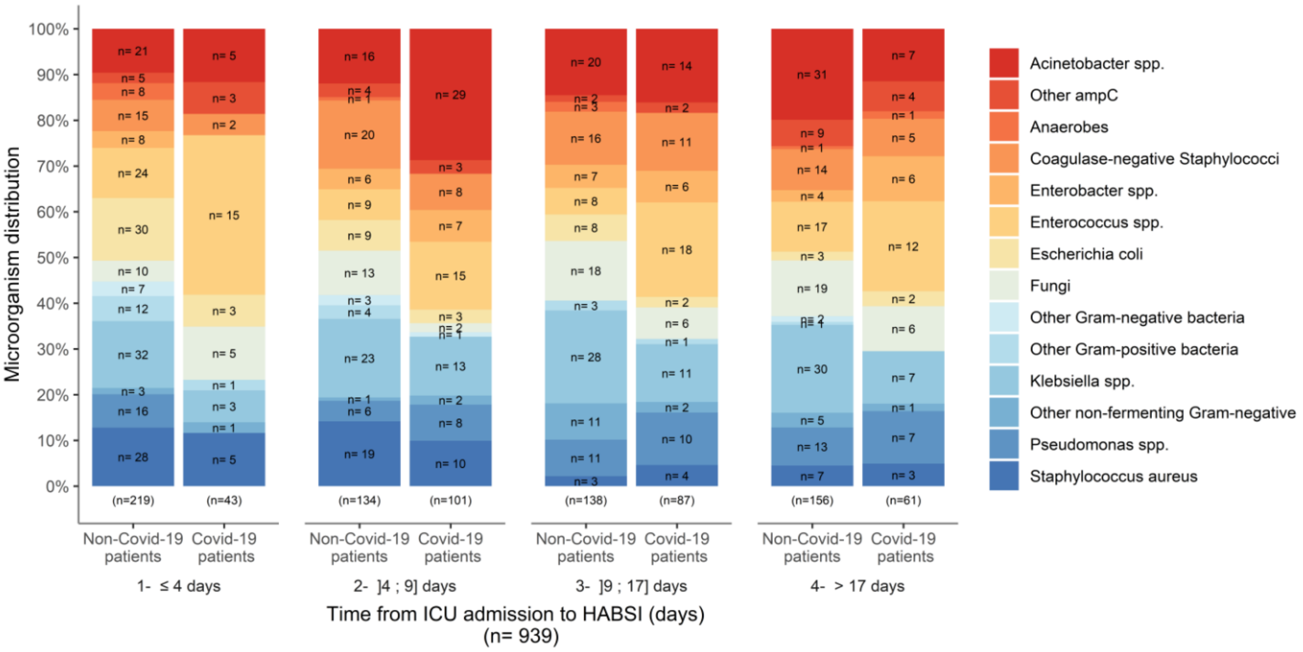

Supplement: Supplementary file 1 — Additional file 1. Additional methods (definitions, additional methods, statistical analyses and ethics), Additional tables (Tables S1–S8) and Additional figures (Figs. S1–S2). [file 13054_2022_4166_MOESM1_ESM.pdf]
